# Multicenter EuroTravNet/GeoSentinel Study of Travel-related Infectious Diseases in Europe

**DOI:** 10.3201/eid1511.091147

**Published:** 2009-11

**Authors:** Philippe Gautret, Patricia Schlagenhauf, Jean Gaudart, Francesco Castelli, Philippe Brouqui, Frank von Sonnenburg, Louis Loutan, Philippe Parola, for the GeoSentinel Surveillance Network,

**Affiliations:** Assitance Publique–Hôpitaux de Marseille, Marseille, France (P. Gautret, P. Brouqui, P. Parola); Zurich University Centre for Travel Medicine, Zurich, Switzerland (P. Schlagenhauf); Faculty of Medicine of Marseille, Marseille (J. Gaudart); University of Brescia, Brescia, Italy (F. Castelli); Ludwig Maximilians Universität of Munich, Munich, Germany (F. von Sonnenburg); Geneva University Hospitals, Geneva, Switzerland (L. Loutan); 1 A list of GeoSentinel Surveillance Network members who also contributed data is given at the end of this article.

**Keywords:** Travel, Europe, imported diseases, infectious diseases, bacteria, viruses, maslaria, dengue, postexopsure prophylaxis, research

## Abstract

We analyzed prospective data on 17,228 European patients who sought treatment at GeoSentinel sites from 1997 to 2007. Gastrointestinal illness (particularly in tourists), fever (those visiting friends and relatives [VFRs]), and skin disorders (in tourists) were the most common reasons for seeking medical care. Diagnoses varied by country of origin, region visited, or categories of travelers. VFRs who returned from sub-Saharan Africa and Indian Ocean islands were more likely to experience falciparum malaria than any other group. Multiple correspondence analysis identified Italian, French, and Swiss VFRs and expatriate travelers to sub-Saharan Africa and Indian Ocean Islands as most likely to exhibit febrile illnesses. German tourists to Southeast and south-central Asia were most likely to seek treatment for acute diarrhea. Non-European travelers (12,663 patients from other industrialized countries) were less likely to acquire certain travel-associated infectious diseases. These results should be considered in the practice of travel medicine and development of health recommendations for European travelers.

In recent years, growth in international travel has been ≈6% per year, and similar trends are expected in the future (*1*). This growth has been strongly driven by travelers to newly popular destinations in Asia and the Pacific, Africa, and the Middle East (*1*). Approximately 80 million persons from industrialized nations travel to the developing world each year, and an estimated >200 million persons now reside outside their country of birth (*1*).

European travelers represent most of the international travelers, with Germany, United Kingdom, France, and Italy the leading countries of origin (*2*). With few exceptions, no European consensus exists on recommendations for travelers about risk assessment, malaria prophylaxis, or vaccinations. International references include the World Health Organization green book (*3*), which emphasizes risk assessment by rates of diseases in local populations; and the Centers for Disease Control and Prevention yellow book (*4*), which examines risk in the context of American travelers. Yet, whether these guidelines are appropriate in the European context is not known.

The intense international traffic between Europe and the rest of the world means that travelers have become a key element in the global spread of infectious diseases. These diseases may be introduced into domestic European populations and environments that are receptive to further spread. In 2003, severe acute respiratory syndrome (SARS) was introduced to France by 1 patient who returned from Vietnam (*5*). Malaria has recently reemerged in Italy and in France (Corsica), resulting from local transmission by anopheline mosquitoes that fed on travelers who had become infected with *Plasmodium vivax* during travel (*6*). More recently, chikungunya virus (CHIKV) appeared as a paradigm of an infectious disease that rapidly became global as highly viremic travelers acted as efficient carriers of the virus (*7*). After CHIKV-infected persons in eastern Africa, Indian Ocean islands, India, and Southeast Asia, a new CHIKV variant reached Europe and affected local populations in Italy through 1 infected traveler (the index case-patient) and transmission by indigenous European mosquito vectors (*8*). In April 2009, an influenza A pandemic (H1N1) 2009 virus emerged in humans in North America and reached Europe soon after through returned travelers (*9*).

European physicians should be prepared to encounter and recognize infectious imported diseases. Facing the symptoms and syndromes in the ill returned traveler requires an understanding of the common etiologic agents encountered by different populations of travelers (*10*). Accurate epidemiologic data are needed about travel-associated infectious diseases in travelers returning to European countries. Some data on diseases among Europeans who traveled to developing countries recently have been published but were limited to 1 country of origin (*11*–*13*), a short period of study, specific diseases (*14*–*16*), a specific destination (*17*), or a certain type of traveler (*18*). A comprehensive multicenter comparison of the spectrum of illnesses among European travelers, including a broad sample of destinations, has been missing. Our objective in this study was to determine the epidemiology of travel-related infectious diseases in a large set of ill returned European travelers over a substantial period and to compare this with the epidemiology of disease in travelers from other industrialized countries outside Europe.

## Patients and Methods

### Data Source

The GeoSentinel Surveillance Network consists of specialized travel/tropical medicine clinics on 6 continents where ill travelers are seen during or after traveling to a wide range of countries and where information about travelers is prospectively recorded (*19*) in a standardized format. To be eligible for inclusion in the GeoSentinel database, patients must have crossed an international border and have received medical attention at a GeoSentinel clinic for a presumed travel-related illness. We included western European patients who sought treatment at GeoSentinel sites after travel from March 1997 through November 2007. Persons were placed in 3 different categories: classic traveler, immigrant traveler, and expatriate traveler (Table 1). Reasons for travel were classified as the following: tourism, business, research/education, missionary/volunteer work, or visiting friends and relatives (VFRs). Individual countries visited were grouped into 12 regions (*19*). Medical data included the final physician-assigned diagnosis, according to a standardized list of 556 possible individual diagnoses of infectious diseases that were also categorized under 21 broad syndromes as previously described (*19*). European patients were compared with all other ill non-European returned patients on the basis of information obtained from GeoSentinel sites in the United States, Canada, Australia, and New Zealand.

**Table 1:** Categories of ill European* returned travelers seen at GeoSentinel sites, 1997–2007

Category	Definition
Classic traveler	European country–born person living in Europe who traveled to a developing country and has returned to his or her home country.
Immigrant traveler	Person born in a developing country who at some time has emigrated to Europe,† including refugees, where a permanent residence has been established, and who later travels to a developing country and returns to Europe.
Expatriate traveler	European-born person who grew up in Europe and whose current country of residence is a developing country. They were included when they sought treatment at a GeoSentinel site after they returned to Europe and/or after travel while still expatriating.

*From Western Europe (*19*). †Patients whose purpose of travel was the initial immigration travel from their birth country to Europe were excluded.

### Statistical Analysis

Data were entered and managed in Microsoft Access (Microsoft Corp., Redmond, WA, USA). In our evaluation, proportionate morbidity refers to the number of cases of a specific diagnosis (or of a group of specific diagnosis within a syndrome group) compared with all cases of ill returned travelers seen at GeoSentinel clinics during the same period. Differences in proportions (qualitative variables) were tested by using Pearson χ^2^ or Fisher exact tests. Analysis of variance or Kruskal-Wallis tests were used for quantitative variables. Because of the large numbers of statistical tests performed, a p value <0.001 was considered significant.

Diagnosis, exposure regions, residence region, and travel types were analyzed by using multiple correspondence analysis (MCA) (*20*–*22*). MCA was performed by using the ANADEV freeware (www.lertim.org), developed by the Laboratory of Biomathematics, Faculty of Medicine of Marseille. Odds ratios (ORs) (European vs. non-European) by diagnosis were estimated by using logistic regression and adjusted for travel duration. All statistical tests were 2-sided. Percentages and odds ratios (with 95% confidence intervals), comparisons, and graphic analysis were carried out by using the R 2.8.1 environment (www.r-project.org).

## Results

### Demographic and Travel Data

**Figure 1:**
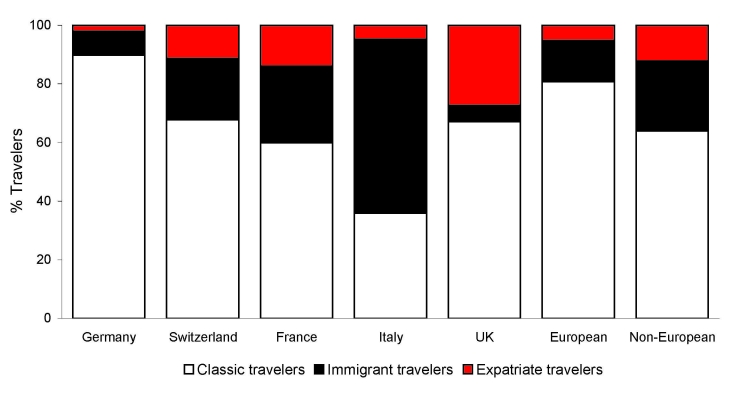
Proportion (%) of different categories of returned patients among 17,228 patients seen in GeoSentinel sites in Europe,* compared with 12,663 non-European patients sampled from the GeoSentinel database (1997–2007). *This proportion includes 11,848 from Germany, 2,818 from Switzerland, 971 from Italy, 931 from France, 289 from the United Kingdom, and 371 from other European countries.

**Figure 2:**
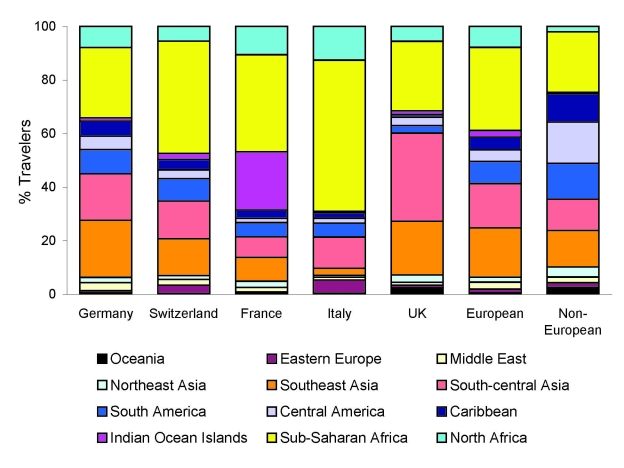
Regions visited by the 17,228 European travelers according to their countries of residence or citizenship. The right column presents these categories within the comparator groups of 12,663 non-European patients sampled from the GeoSentinel database (1997–2007).

A total of 17,228 European patients were included: 13,913 (80.8%) classic travelers, 2,415 (14.0%) immigrant travelers, and 900 (5.2%) expatriate travelers (Figure 1). Demographic and travel data are presented in Table 2. Most patients were seen as outpatients who sought treatment at the clinics <2 weeks post travel. Immigrant travelers sought markedly less pretravel advice and were more likely to be inpatients than other groups; differences were significant (p<0.0001). Furthermore, European patients’ main destination was Africa, followed by Asia; the proportion of patients returning from sub-Saharan Africa, Indian Ocean islands, and south-central Asia was higher in sites in Italy, France, and the United Kingdom, respectively (Figure 2). Non-Europeans (12,663 patients) had a lower proportion of immigrant travelers in the inpatient category, and non-European expatriates were younger, had a longer duration of travel, and sought pretravel advice more often (p<0.0001).

**Table 2:** Demographic and travel data for 17,228 European travelers seen at GeoSentinel European sites, compared with non-European travelers, 1997–2007*

Characteristic	European				Non-European†		
	Classic travelers	Immigrant travelers	Expatriate travelers		Classic travelers	Immigrant travelers	Expatriate travelers
Sex							
M	6,882 (49.5)	1,440.(59.6)	526 (58.4)		3,608 (44.5)	1,513 (50.2)	1,006 (64.9)
F	7,006 (50.4)	971 (40.2)	372 (41.3)		4,278 (52.8)	1,463 (48.6)	532 (34.3)
Unknown	25 (0.2)	4 (0.2)	2 (0.2)		215 (2.7)	37 (1.2)	11 (0.7
Median age, y (range)	34 (0–96)	36 (0–95)	37 (1–179)		32 (0–95)	40 (2–89)	23 (1–77)
Patient type							
Inpatient	611 (4.4)	887 (36.7)	65 (7.2)		436 (5.4)	323 (10.7)	53 (3.4)
Outpatient	13,243 (95.2)	1,507 (62.4)	785 (87.2)		7,233 (89.3)	2,595 (86.1)	1,448 (93.4)
Unknown	59 (0.4	21 (0.9	50 (5.6)		432 (5.3)	95 (3.2)	48 (3.1)
Median travel duration,‡ d (range)	21 (1–;212)	30 (1–180)	180 (1–1,555)		20 (1–212)	26 (1–198)	334 (1–1,010)
Pretravel advice							
Yes	8,212 (59.0)	525 (21.7)	518 (57.6)		4,169 (51.5)	647 (21.5)	1,209 (78.1)
No	3,252 (23.4)	1,206 (49.9)	186 (20.7)		2,678 (33.1)	1,927 (64.0)	216 (13.9)
Unknown	2,449 (17.6)	684 (28.3)	196 (21.8		1,254 (15.5	439 (14.6)	124 (8.0)
Reason for travel							
Tourism	11,200 (80.5)	525 (21.7)	124 (13.8)		5,317 (65.6)	813 (27.0)	81 (5.2)
Business	1,733 (12.5)	142 (5.9)	344 (38.2)		1,084 (13.4)	216 (7.2)	273 (17.6)
Missionary or volunteer work	775 (5.6)	59 (2.4)	422 (46.9)		1,225 (15.1)	137 (4.5)	1,190 (76.8)
Student	82 (0.6)	23 (1.0)	2 (0.2)		355 (4.4)	92 (3.1)	–
Healthcare seeking	8 (0.1)	–	–		–	1 (0.1)	–
Visiting friends or relatives	100 (0.7)	1,662 (68.8)	8 (0.9)		96 (1.2)	1,752 (58.1)	5 (0.3)
Military	15 (0.1)	4 (0.2)	124 (13.8)		24 (0.3)	2 (0.1)	–
Median time between travel end and presentation, d (range)	13 (1–156)	13 (1–139)	10 (1–153)		17 (1–154)	26 (1–157)	23 (1–165)

*All values are no. (%) except as indicated. For each demographic and travel variable, univariate global analyses were used for comparing means (analysis of variance) or percentages (χ^2^ test). For example, age means (for each category) have been compared using an analysis of variance; percentages of men (for each category) have been compared by χ^2^ test. All tests have shown significant results (p<0.0001). †Data for 12,663 non-European patients sampled from the GeoSentinel database. ‡Travel duration and interval time were estimated using accurate data, available for 15,969 (92.7%) Europeans and 9,828 (77.6%) non-Europeans exhibiting a travel-associated disease along with a recent trip (<6 mo ago).

### Final Etiologic Diagnosis

The proportionate morbidity of some broad syndromes or etiologic diagnoses was higher in patients travelling to specific regions. This was obvious for acute diarrhea in North Africa, south-central Asia, and the Middle East, and etiologic diagnosis such as *Campylobacter* spp. in south-central and Southeast Asia, *Shigella* spp. in North Africa and south-central Asia, *Giardia* spp. in south-central Asia and South America and amebas in south-central Asia. Febrile systemic illnesses were more frequently reported from Indian Ocean islands, sub-Saharan Africa, and Oceania. *P. falciparum* malaria was more frequently observed in travelers returning from Indian Ocean islands and sub-Saharan Africa, *P. vivax* malaria in travelers from Oceania, Indian Ocean islands, and South America, and *P. ovale* and *P. malaria*e malaria in travellers from Indian Ocean islands and sub-Saharan Africa. Dengue was more frequently reported from Southeast Asia, chikungunya from Indian Ocean Islands, rickettsioses from sub-Saharan Africa, and salmonellosis from south-central Asia. Proportionate morbidity for dermatologic conditions was higher in Oceania, Southeast Asia, Central America, South America, and the Caribbean, including animal-related injuries requiring rabies postexposure prophylaxis (PEP) in North Africa, the Middle East, and Southeast Asia; larva migrans in Southeast Asia, the Caribbean, South America, and Central America; leishmaniasis in Central America and South America; and myasis in Central America. Finally, respiratory syndromes were more frequently reported in travelers returning from eastern Europe and northeastern Asia; genitourinary and sexually transmitted diseases (STDs) were more frequent in travelers from eastern Europe, Southeast Asia, and the Caribbean; schistosomiasis was more frequent in travellers from Africa and cerebromeningeal infections were more frequent in travelers from eastern Europe and North Africa) (p<0.0001) (Technical Appendix).

Also, the proportionate morbidity of some broad syndromes or etiologic diagnoses was higher in persons returning to specific European countries, as illustrated for falciparum malaria (Italy, France), dengue (United Kingdom), CHIKV infection (France), animal-related injuries requiring rabies PEP (France, United Kingdom) and cerebromeningeal infections (Italy) (p<0.0001). The proportionate morbidity was also higher in some categories of traveler, such as diarrhea and dermatologic diseases (in classic tourist travelers), falciparum malaria and genitourinary infections and STDs (immigrant travelers who were VFRs), and *P. vivax* malaria (missionary/expatriate travelers) (p<0.0001). (For details, see Technical Appendix.)

MCA highlights the possibility of diagnosis in certain groups and shows an association between German patients, who are classic travelers (traveling for tourism to Southeast and south-central Asia) and a diagnosis of acute diarrhea. The MCA also showed that French, Swiss, or Italian patients who are classified as immigrant or expatriate travelers (VFRs or travelers for missionary purposes to Africa or Indian Ocean islands) are most likely to seek treatment for febrile illness (Technical Appendix).

**Figure 3:**
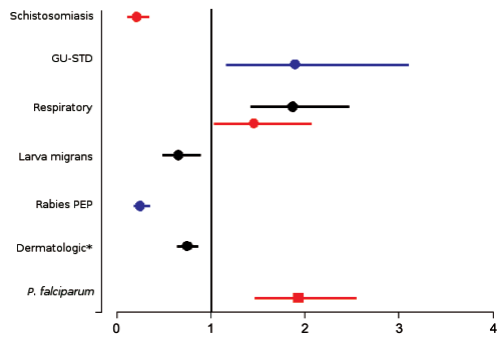
Odds ratios (ORs), European (n = 13,488) versus non-European (n = 6,900), by diagnosis and type of ill traveler, adjusted for travel duration. Each plot represents the estimated OR, and 95% confidence intervals are presented by lines. Only significant ORs based on the comparison of groups of >75 ill patients given a diagnosis are shown. The 3 main exposure regions are presented (Africa and Indian Ocean islands (red dots and squares), South and Central America and Caribbean (black dots), Southeast and south-central Asia (blue dots). *All dermatologic diagnoses also include leishmaniasis, animal-related injuries requiring rabies post-exposure prophylaxis (PEP), and larva migrans. GU-STD, genitourinary and sexually transmitted diseases. Dots, classic tourist travelers; square, immigrant travelers visiting friends and relatives (VFRs).

Compared with the corresponding proportion of disease in non-European travelers, European classic tourist travelers had a lower proportionate morbidity (adjusted for travel duration) for certain diagnoses, such as schistosomiasis, cutaneous larva migrans, and animal-related injuries requiring rabies PEP, and a higher proportionate morbidity for genitourinary infections, STDs, and respiratory diseases when traveling to specific regions (Figure 3). Also, the *P. falciparum* malaria proportionate morbidity in immigrant travelers (VRFs) after travel to Africa or the Indian Ocean islands was higher in Europeans compared with non-Europeans (Figure 3).

## Discussion

Despite the large number of patients investigated here in Europe for the assessment of travel-related illness, our work does not analyze all infectious illness in all returned patients. The results do not represent the broad spectrum of illness typically seen at nonspecialized primary care practice where mild or self-limited conditions would be found with higher frequency (*19*,*23*). The intake at sites reflects a mixed population of tertiary care and self-referred patients. Diagnoses that may be underrepresented include diseases of short incubation, many cases of which manifest during travel. However, GeoSentinel captures a sentinel sample of travelers; we have no reason to believe that cases we have not captured would have a different pattern of geographic acquisition than those in GeoSentinel. Also, we cannot relate our data collected on ill travelers to the total number of travelers to or from the area concerned. Because of this absence of denominator, incidence rates cannot be calculated or a numerical risk provided for travel to a particular destination. Absolute risk can be estimated only by monitoring cohorts prospectively, as was conducted in a few studies in the 1980s. Relatively small sample sizes and the limited number of destinations visited by travelers originating in 1 country are usually insufficient to elucidate destination-specific risk for individual diagnoses. Risk also could be calculated from the rate of illness in all travelers to each destination. However, capturing data on all ill travelers to just 1 destination, or even accurately ascertaining the denominator of all travelers to that destination, is not easily accomplished. No published studies have been able to describe this approach on a multicountry or worldwide basis.

However, given these caveats, the major strengths of our analysis are its focus on proportionate disease and the large numbers of patients in the database, which reduces the population-specific bias found in many smaller studies. Important published studies on several aspects of travel medicine have used the GeoSentinel database, now identified as a main source for the epidemiology of travel-related illness (*18*,*19*,*24*–*27*). We selected and discussed specific syndromes and their causes. The European aspect of our study is unique.

Most patients in our survey were outpatients. Ubiquitous or cosmopolitan infections involving the skin and the respiratory, gastrointestinal, and urinary tracts were found frequently in our study as were imported tropical diseases (although the specific tropical/cosmopolitan disease ratio cannot be calculated accurately because etiolgoc agents were not systematically identified or recorded). As previously emphasized, healthcare providers should not overlook such cosmopolitan infections when examining patients returning from the tropics (*28*). Overall, of 10 ill European returned travelers, 4 had a gastrointestinal disorder, 2 experienced a febrile systemic illness, 2 sought treatment for a dermatologic problem, and 1 had a respiratory disease. Acute diarrhea is the most common travel-associated disease (*10*), and we show here that some destinations are more frequently associated with some specific causes. Also, all categories of European travelers to North Africa, south-central Asia, and the Middle East (but particularly classic tourist travelers) should be targeted for pretravel advice regarding diarrhea risk and self-treatment (*29*). Furthermore, the importance of respiratory diseases in travelers has been exemplified with clusters of measles after importation (*30*), and more recently, the emergence and global spread of influenza A pandemic (H1N1) 2009 virus (*9*). Moreover, seasonal influenza, which affects 5%–15% of the world's population annually and has been considered the second most frequent vaccine-preventable infection in travelers, is probably underestimated in returned travelers (*31*).

We highlight here that malaria remains the most common specific diagnosis in ill returned patients who have a systemic febrile illness (*23*). *P. falciparum* was the most commonly identified malaria species causing these infections, which mirrors situation in sub-Saharan Africa, a major source of malaria for European ill returned patients (*32*). The risk to travelers of acquiring malaria varies by destination. However, as shown here, the traveler profile also is an important determinant of malaria risk. *P. falciparum* malaria is a rare diagnosis among native Germans traveling for tourism but it is a frequent diagnosis among immigrant travelers from Italy and France who visit friends and relatives in sub-Saharan Africa and the Indian Ocean islands. As shown here, immigrant travelers (VFRs) rarely seek pretravel advice, and they are known to comply poorly with malaria chemoprophylaxis (*32*). Therefore, immigrant travelers represent a major group at risk for imported malaria in Europe, and an improved approach to educate this population about risks and prophylaxis needs to be developed.

Dengue is now considered one of the major causes of fever in ill returned travelers, who even may serve as important sentinels of new outbreaks of dengue in dengue-endemic areas (*33*). Here, dengue virus was the second most commonly identified pathogen responsible for fever, particularly in patients who returned from Southeast Asia. The incidence of dengue has been considered to be higher than that of other so-called typical travel-related diseases, such as vaccine-preventable hepatitis A and typhoid fever (*34*). Because of rapid, intercontinental transportation, European physicians now encounter patients with arbovirus infections that have short incubation periods, such as dengue, and patients who are still viremic. These factors raise the possibility of introducing the virus to non–dengue-endemic areas where competent vectors are prevalent, as was demonstrated for CHIKV in 2007 (*7*).

Some aspects described here may also influence medical practice that affects returned patients. For example, enteric fever caused by *Salmonella* infection was mainly observed in patients returning from south-central Asia, where multidrug resistance has been established and fluoroquinolone resistance is increasing (*35*).

Our results show the increasing importance of rickettsioses in ill returned travelers, particularly African tick-bite fever, which affects travelers to sub-Saharan Africa, especially those who go on safari and military personnel. These groups of travelers need to be singled out to receive advice on tick-bite prevention (*36*).

Our study also reinforces the view that dermatologic conditions are a leading cause of health problems in travelers (*37*). Pretravel advice should support the traveler’s use of impregnated bed nets and repellents, promote the practice of efficient clothes drying and ironing to prevent myasis, and discourage direct contact of skin with wet soil to prevent larva migrans transmission.

Notably, a larger numbers of patients seeking rabies PEP were observed in France and the United Kingdom, where GeoSentinel clinics include rabies treatment centers. This highlights the potential for rabid animal–related injury in travelers, particularly in North Africa and the Middle East (*24*).

German ill travelers were overrepresented in our collective database because of the historical development of GeoSentinel and the predominance of Germans among European travelers. Furthermore, each GeoSentinel site has specific characteristics, and some would be considered as sentinel sites for diseases in specific categories of travelers returning from particular countries. For example, at the site in Marseille, France, the French colonial past has a large effect on the profile of imported disease. The city has the largest community of inhabitants from the Comoros Islands, Indian Ocean, including first- to third-generation migrants. Immigrant travelers (VFRs) from the Comoros Islands are major importers of *P. falciparum* malaria and were key to creating the initial alert about the CHIKV disease outbreak (*38*).

Differences in disease patterns between countries of origin may reflects national differences in the characteristics of the traveling population, the distribution of travel destinations, and referral and access to medical care. In addition, accommodation standards, eating habits, and other risk behavior at a given destination may reflect the national and cultural background of the traveler. These circumstances also apply when comparing European and non-European returned patients. However, although the non-European comparative group is heterogeneous, the diversity allows us to highlight some characteristics of European travel-related illnesses, such as the falciparum malaria within immigrant travelers (VFRs) in sub-Saharan Africa and the Indian Ocean islands. The economic situation of immigrants in Europe is unlikely to be as secure as that of second- or third-generation immigrants living in the United States, even if they have an easy access to the health system, including university hospitals in many cities. These factors, together with a higher likelihood of having severe imported diseases, such as malaria, may explain the high rate of immigrant travelers (VFRs) who were hospitalized. In Marseille, most of the immigrant travelers originating from Comoros claimed that some types of antimalarial chemoprophylaxis are too expensive for a whole family who travels every 2 years to visit friends and family.

European and non-Europeans ill returned travelers may also have a different code of conduct and behavior. For example, classic tourist travelers from Europe to Asia have a higher proportion of STDs than do other travelers. Again, our ill travelers probably do not reflect the whole population of travelers returning from the tropics with STDs because many probably consult their general practitioners first. However, a broad spectrum of STDs recently have been highlighted as common causes of health impairment among European travelers returning from the tropics, and Asia has destinations known for sex tourism (*39*).

Furthermore, depending on the destination, tourist travelers seem to be less frequently afflicted by diseases transmitted by contact of skin with fresh water or wet soil (schistosomiasis and larva migrans) and interaction with animals (animal-related injuries requiring rabies PEP); these facts suggest that they may be more compliant with travel health recommendations. We have no clear explanation, however, for the higher respiratory disease–related illnesses for European tourists traveling to Africa and America, but we note that SARS was imported to Europe in this way.

## Conclusions

Clinicians encountering returned patients have an essential role in recognizing, and communicating travel-associated public health risks (*19*,*23*). In this context, surveillance in European travelers that encompasses a wide range of sites in Europe, including some with local specificity, is crucial to determine the epidemiology of travel-associated disease, to detect alarming events, and, if required, to organize a rapid response (*40*). Our combined European data can be used as background evidence for the practice of travel medicine in Europe.

## Supplementary Material

Technical Appendix Selected etiologic diagnosis within selected syndrome groups, according to countries of residence or citizenship and to travel region among 17,228 European travelers seen at GeoSentinel sites, 1997-2007*daggar, double daggar
