# Supplementary material for: Multicenter EuroTravNet/GeoSentinel Study of Travel-related Infectious Diseases in Europe
Source: Emerg Infect Dis. 2009 Nov;15(11):1783–90. doi: 10.3201/eid1511.091147 (PMC2857260; doi:10.3201/eid1511.091147)
Supplement: Technical Appendix — Selected etiologic diagnosis within selected syndrome groups, according to countries of residence or citizenship and to travel region among 17,228 European travelers seen at GeoSentinel sites, 1997-2007*daggar, double daggar [file 09-1147_Techapp-s1.pdf]

Multicenter EuroTravNet/GeoSentinel Study of Travel-related Infectious Diseases in Europe

Technical Appendix

Table 1. Selected etiologic diagnosis within selected syndrome groups, according to countries of residence or citizenship and to travel region among 17,228 European travelers seen at GeoSentinel sites, 1997–2007*†‡

	Country of residence or citizenship						Travel region											
Diagnosis	Germany	Switzerland	Italy	France	UK	Europe (total)	North Africa	Sub-Saharan Africa	Indian Ocean Islands	Caribbean	Central America	South America	South-Central Asia	Southeast Asia	Northeast Asia	Middle East	Eastern Europe	Oceania
No. patients	11,848	2,818	971	931	289	17,228	1,343	5,349	415	825	742	1,436	2,850	3,158	323	452	245	90
Acute diarrhea§	23.9	23.6	12.2	13.0	25.3	22.7	35.2	16.4	13.0	21.0	22.0	22.0	34.3	19.2	20.4	30.5	23.7	6.7
Febrile systemic illness	18.9	25.9	51.0	36.0	21.5	23.0	8.9	33.2	55.2	20.0	15.4	16.3	16.9	22.3	14.6	8.0	10.2	33.3
P. falciparum malaria	1.5	7.7	30.9	21.2	2.4	5.3	0.3	13.9	32.0	0.6	0.0	0.3	0.2	0.4	0.0	0.0	0.0	0.0
Malaria, other	1.1	2.9	4.1	4.1	3.8	1.8	0.4	3.1	5.8	0.1	0.8	1.9	0.9	1.1	0.0	0.0	0.4	14.4
Dengue	2.5	1.8	1.9	2.3	3.8	2.4	0.2	0.7	1.9	3.5	3.8	2.6	3.1	5.7	0.0	0.0	0.8	6.7
Chikungunya	0.1	0.4	0.2	1.7	0.3	0.2	0.0	0.1	6.3	0.0	0.0	0.0	0.4	0.1	0.0	0.0	0.0	0.0
Rickettsioses	0.7	0.4	0.2	0.2	0.3	0.6	0.2	1.7	0.2	0.1	0.1	0.0	0.1	0.2	0.0	0.4	0.0	0.0
Salmonelloses	0.1	0.7	0.9	0.1	1.7	0.3	0.1	0.2	0.2	0.2	0.0	0.3	1.4	0.1	0.0	0.0	0.0	0.0
Dermatologic	17.1	12.5	7.7	21.1	15.6	15.9	13.8	12.4	14.9	20.4	21.2	20.7	13.5	21.4	7.4	14.2	15.1	30.0
Respiratory	7.5	5.8	9.6	4.0	10.0	7.3	4.3	7.4	1.9	7.2	7.3	6.0	7.7	8.5	12.7	4.9	15.1	4.4
GU-STD	2.9	2.9	4.9	2.4	4.2	3.0	2.5	3.1	1.4	3.4	1.8	3.0	2.9	3.5	2.2	2.9	4.9	1.1
Schistosomiasis	0.5	2.1	1.5	0.9	2.1	0.9	1.0	2.0	1.2	0.1	0.1	0.7	0.2	0.3	0.0	0.4	0.0	0.0
Cerebromenigeal infection	0.2	0.4	2.5	0.2	0.7	0.3	1.0	0.2	0.2	0.4	0.1	0.5	0.2	0.3	0.0	0.4	2.4	0.0

**P.*, *Plasmodium*; GU-STD, genitourinary and sexually transmitted diseases.
†Related morbidity percentage of patients with a specific diagnosis, or group of diagnosis as a proportion of all ill returned travelers.
‡For etiologic diagnosis within acute diarrhea, non–P. falciparum malaria, and dermatologic syndromes, see Table 2.
§<2 wk.

Table 2. Selected etiologic diagnosis within categories of acute diarrhea (<2 wks), dermatologic syndrome, and non-falciparum malaria groups, according to countries of residence or citizenship and to travel region among 17,228 European travelers, seen at GeoSentinel sites, 1997–2007*†

	Country of residence or citizenship						Travel region											
Diagnosis	Germany	Switzerland	Italy	France	UK	Europe (total)	North Africa	Sub-Saharan Africa	Indian Ocean Islands	Caribbean	Central America	South America	South-Central Asia	Southeast Asia	Northeast Asia	Middle East	Eastern Europe	Oceania
No. patients	1,1848	2,818	971	931	289	17,228	1,343	5,349	415	825	742	1,436	2,850	3,158	323	452	245	90
Acute diarrhea	23.9	23.6	12.2	13.0	25.3	22.7	35.2	16.4	13.0	21.0	22.0	22.0	34.3	19.2	20.4	30.5	23.7	6.7
Campylobacter	2.8	0.7	0.4	0.1	0.7	2.2	1.8	1.2	1.2	1.1	0.9	2.2	3.8	3.5	0.3	1.8	2.4	0.0
Shigella	1.2	1.0	0.3	0.5	0.3	1.1	2.2	0.7	1.0	1.1	0.1	1.3	2.0	0.4	0.3	1.8	0.4	0.0
Salmonella NT	1.0	0.7	0.7	1.1	1.0	1.0	1.3	0.7	0.7	1.0	0.5	0.7	1.0	1.6	0.6	1.3	2.9	0.0
Giardia	4.6	5.1	1.4	2.0	5.2	4.3	2.7	2.9	1.2	2.5	2.8	5.0	11.4	2.6	1.5	2.4	2.9	1.2
Amebas	1.4	1.6	0.7	1.4	0.7	1.4	1.4	1.3	0.5	1.2	2.2	1.5	2.1	1.0	1.2	1.3	2.4	0.0
Dermatologic	17.1	12.5	7.7	21.1	15.6	15.9	13.8	12.4	14.9	20.4	21.2	20.7	13.5	21.4	7.4	14.2	15.1	30.0
Rabies PEP	0.5	1.4	0.0	12.2	5.5	1.4	4.3	0.5	0.5	0.4	0.9	0.7	0.9	2.8	1.9	3.3	2.9	0.0
Bite arthopods	4.6	2.3	0.8	1.1	1.0	3.7	2.4	4.6	4.6	5.5	5.3	4.9	3.4	5.1	1.2	2.2	4.5	10.0
Larva migrans	1.9	1.4	1.0	1.2	1.7	1.8	0.4	0.5	0.5	3.3	2.6	3.2	0.9	3.3	0.3	0.0	0.0	1.1
Bacterial	3.1	2.7	2.6	3.7	2.8	3.1	1.8	4.6	4.6	2.9	3.1	3.3	3.1	4.1	0.9	2.7	3.7	10.0
Leishmaniasis	0.2	0.1	0.7	0.2	0.0	0.2	0.7	0.0	0.0	0.0	0.7	0.7	0.2	0.0	0.0	0.9	0.4	0.0
Myasis	0.2	0.5	0.0	0.4	0.0	0.3	0.0	0.2	0.2	0.0	2.0	0.8	0.0	0.0	0.0	0.0	0.0	1.1
Non–*P. falciparum* malaria	1.1	2.9	4.1	4.1	3.8	1.8	0.4	3.1	5.8	0.1	0.8	1.9	0.9	1.1	0.0	0.0	0.4	14.4
P. vivax	0.5	1.1	2.5	2.0	2.8	0.8	0.2	0.7	3.4	0.1	0.5	1.7	0.7	0.8	0.0	0.0	0.0	13.3
P. ovale	0.1	0.6	0.9	1.0	0.0	0.3	0.1	0.7	1.0	0.0	0.0	0.1	0.1	0.1	0.0	0.0	0.0	0.0
P. malariae	0.0	0.3	0.7	0.4	0.0	0.1	0.1	0.4	0.7	0.0	0.0	0.0	0.0	0.0	0.0	0.0	0.0	0.0

**P.*, *Plasmodium*; NT, non-typhi; PEP, postexposure prophylaxis.
†Related morbidity percentage of patients with a specific diagnosis or group of diagnoses as proportion of ill returned travelers.

Table 3. Selected etiologic diagnoses within selected syndrome groups and selected diseases among and 17,228 European travelers according to categories of travelers* seen at GeoSentinel sites, 1997–2007†

Diagnosis	Classic tourist	Immigrant VFR	Missionary expatriate	Expatriate business
Acute diarrhea (<2 wk)	10.5	26.0	20. 3	20.1
Febrile systemic illness	19.2	44.6	20.3	21.2
Plasmodium falciparum	1.5	24.8	5.0	4.4
P. vivax	0.7	1.0	1.6	1.0
Dermatologic	18.3	11.1	13.3	11.1
GU-STD	2.8	4.3	3.5	2.3
Cerebromeningeal infections	0.3	1.0	0.1	0.1

*Related morbidity percentage of patients with a spcific diagnosis or group of diagnoses as proportion of ill returned travelers.
†VFR, visiting friends and relatives; GU-STD, genitourinary and sexually transmitted diseases.

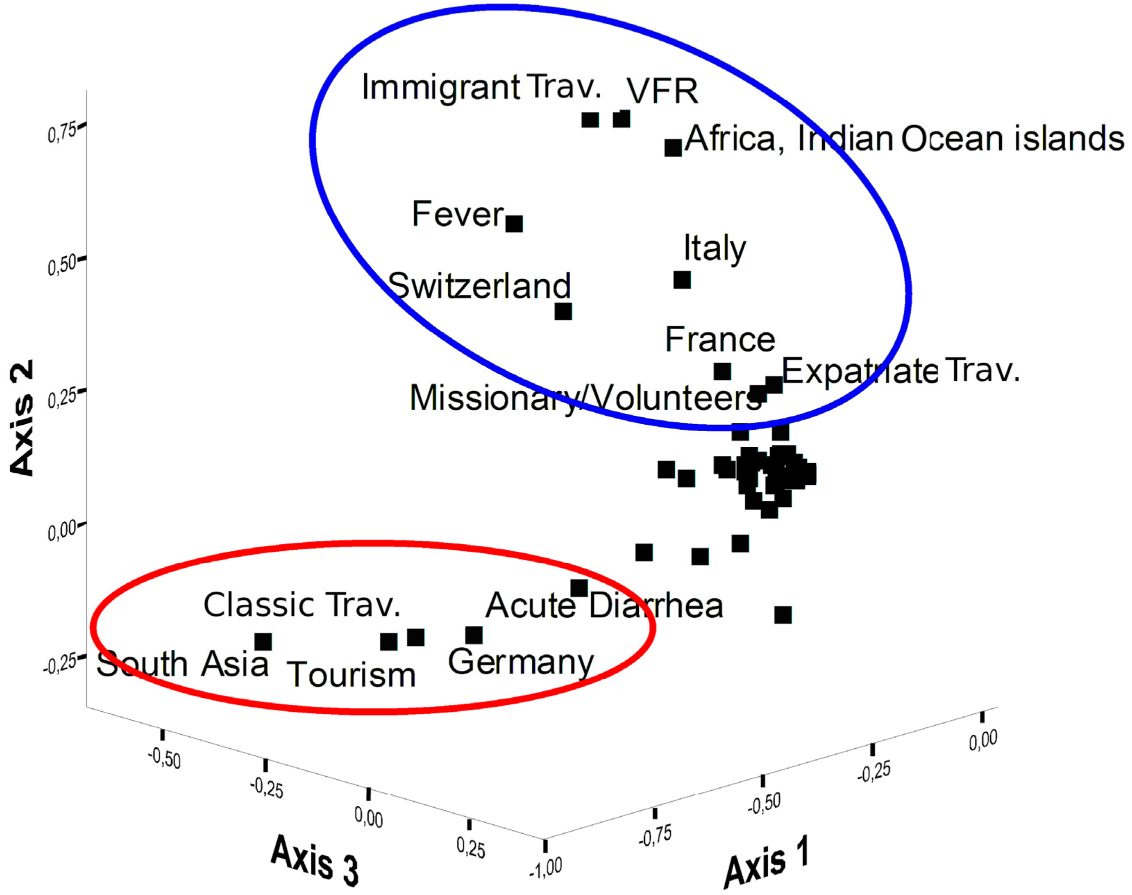

Figure. Representation of the relationships between travel and demographic data within 17,228 European travelers, using multiple correspondence analysis (MCA).* Two groups of data with strong relationships are shown (red and blue circles). The first group (red) shows a relationship for German origin, classic traveler category, tourism as a purpose of travel, with travel to southern Asia and a diagnosis of acute diarrhea. The second group (blue) shows a relationship between immigrant travelers of Italian, French, and Swiss origin, with visiting friends and relatives (VFR) as the purpose of travel, travel to Africa and Indian Ocean Islands, and a diagnosis of fever. *MCA analyzes multiway tables containing some measure of relationship (correspondences) between the rows and columns. It generates graphical representations of the relationship between modalities of categorical variables, and allows the visual discovery and interpretation of this relationship. The correspondence analysis used here presence/absence based distances, which yield a multidimensional representation of the different characters (22-24). The MCA with presence/absence based distances uses positive occurrences (presence of characters) when negative occurrences (absence of characters) are not filled in an equivalent manner. In the GeoSentinel database, the presence of information on a particular symptom provides more information than the lack of information on this symptom.
